# A Novel Approach to Isolating Improved Industrial Interspecific Wine Yeasts Using Chromosomal Mutations as Potential Markers for Increased Fitness

**DOI:** 10.3389/fmicb.2018.01442

**Published:** 2018-07-03

**Authors:** Jennifer R. Bellon, Christopher M. Ford, Anthony R. Borneman, Paul J. Chambers

**Affiliations:** ^1^The Australian Wine Research Institute, Adelaide, SA, Australia; ^2^School of Agriculture, Food and Wine, The University of Adelaide, Adelaide, SA, Australia

**Keywords:** interspecific wine yeast hybrids, evolving populations, chromosomal mutations, increased fitness, retain desired phenotype

## Abstract

Wine yeast breeding programs utilizing interspecific hybridization deliver cost-effective tools to winemakers looking to differentiate their wines through the development of new wine styles. The addition of a non-*Saccharomyces cerevisiae* genome to a commercial wine yeast can generate novel phenotypes ranging from wine flavor and aroma diversity to improvements in targeted fermentation traits. In the current study we utilized a novel approach to screen isolates from an evolving population for increased fitness in a *S. cerevisiae* × *S. uvarum* interspecific hybrid previously generated to incorporate the targeted phenotype of lower volatile acidity production. Sequential grape-juice fermentations provided a selective environment from which to screen isolates. Chromosomal markers were used in a novel approach to identify isolates with potential increased fitness. A strain with increased fitness relative to its parents was isolated from an early timepoint in the evolving population, thereby minimizing the risk of introducing collateral mutations and potentially undesirable phenotypes. The evolved strain retained the desirable fermentation trait of reduced volatile acidity production, along with other winemaking traits of importance while exhibiting improved fermentation kinetics.

## Introduction

An increasingly competitive global market requires winemakers to minimize production costs and target market niches by differentiating their wines through, for example, development of novel wine styles. One way of achieving these ends is to generate new yeast strains with improved fermentation traits and/or novel phenotypes that shape wine flavor and aroma. Such yeasts provide winemakers with tools that are readily and easily introduced into the winery without incurring additional costs or requiring processing interventions.

There are various ways to generate new yeast strains, including breeding programs. Traditionally this would involve mating strains of the same species, which, in the context of wine yeast, is *Saccharomyces cerevisiae*. However, more recently the potential to bring a higher level of phenotypic diversity into wine yeast by hybridization with non-*S. cerevisiae* species of the *Saccharomyces* clade has been realized. This has led to the generation of interspecific hybrids for use in a range of beverage industries, particularly brewing (Krogerus et al., 2015, 2017) and winemaking (Rainieri et al., 1999; Bellon et al., 2011; Pérez-Través et al., 2012). Interspecific hybrids have, for example, incorporated phenotypes from the non- *S. cerevisiae* parent that are either not present in wine yeast, or for which wine yeast has a reduced capacity, respectively: growth at low temperatures (Libkind et al., 2011) and increased glycerol production (Rainieri et al., 1999; González et al., 2007).

Our laboratory previously reported the ability of laboratory-generated interspecific yeast hybrids to introduce flavor and aroma diversity to wines by incorporating the genome of a closely-related *Saccharomyces* species (*Saccharomyces paradoxus* and *Saccharomyces kudriavzevii*) with a commercial *S. cerevisiae* wine yeast strain (Bellon et al., 2011). Utilizing a more divergent *Saccharomyces* species (*Saccharomyces mikatae*) as a genetic contributor generated hybrid strains capable of producing novel, not previously recognized, flavor-active metabolites in wines (Bellon et al., 2013). The most recent publication from this work described the targeted improvement of reduced volatile acidity production in high-sugar fermentation by the generation of *S. cerevisiae* × *S. bayanus* (var. *S. uvarum*) interspecific hybrids (Bellon et al., 2015). The genomes of the *S. cerevisiae* × *S. uvarum* hybrids were shown to be genetically stable following 200 mitotic generations in laboratory nutrient liquid medium as no loss of chromosome from either parent was identified in any of the 20 isolates from each hybrid investigated and no change in the triploid DNA fluorescence levels was observed. One hybrid from this work (AWRI 1572) showed exceptional promise in terms of what it could bring to wine quality (Bellon et al., 2015) and was chosen for further development.

In research from other laboratories the genetic composition of a number of naturally occurring interspecific hybrid strains isolated from different fermentation sources has been evaluated revealing substantial loss of chromosomal material from one or both parental lineages (Dunn and Sherlock, 2008; Borneman et al., 2011, 2016; Peris et al., 2012). This loss may have been due to genome incompatibilities (Scannell et al., 2006), and presumably led to greater fitness for the yeast in the industrial environment they were isolated from.

With this in mind, it was decided to passage the aforementioned laboratory-generated interspecific hybrid wine yeast, AWRI 1572, through a series of successive grape must fermentations with the aim of selecting for spontaneous mutants with increased fitness in a winemaking context. The rationale was to screen for chromosomal mutations in isolates from the evolving passaged populations. As it was not known specifically which chromosomal mutations might improve fitness, chromosomal markers for both arms of all 16 chromosomes from each parent were employed. Any isolates with chromosomal mutations that became highly represented in the populations were candidates for strain development and were subsequently tested for their fitness compared to the AWRI 1572 and for retention of desirable traits of the parent. The potential of this novel approach of screening for highly represented chromosomal mutations as a marker for increased fitness was realized: a chromosomal marker that increased in frequency in the evolving population was identified. This enabled isolation from an early stage of the evolutionary process progeny with increased fitness relative to its parent. This novel hybrid retained the previously reported desirable ‘low acetic acid’ phenotype, along with other winemaking traits of importance.

## Materials and Methods

### Yeast Strains

*Saccharomyces* spp. interspecific hybrid strain AWRI 1572, generated using rare-mating hybridization between *S. cerevisiae* diploid strain (AWRI 838) and spores from *Saccharomyces uvarum* strain AWRI 1176 as described in Bellon et al. (2015); hybrid strain AWRI 2530 (an evolved strain of AWRI 1572 generated in this study) and control yeast strains for haploid, diploid and tetraploid DNA intensity determinations BY4741 *MAT***a**, BY4743 (Euroscarf^®^, Frankfurt, Germany) and 53-7 (Salmon, 1997) respectively.

The *S. uvarum* parent of the hybrid AWRI 1572 had been molecularly typed as *Saccharomyces bayanus* var. *uvarum*; a sub group of the *S. bayanus* species. Recent studies in other laboratories indicate that this sub group should constitute a separate species *Saccharomyces uvarum* (Libkind et al., 2011; Nguyen et al., 2011) thus we refer to AWRI 1176 as *S. uvarum* in this manuscript.

### Selection for Increased Fitness in a Passaged, Evolving Population of Wine Yeast Hybrid AWRI 1572

**FIGURE 1:**
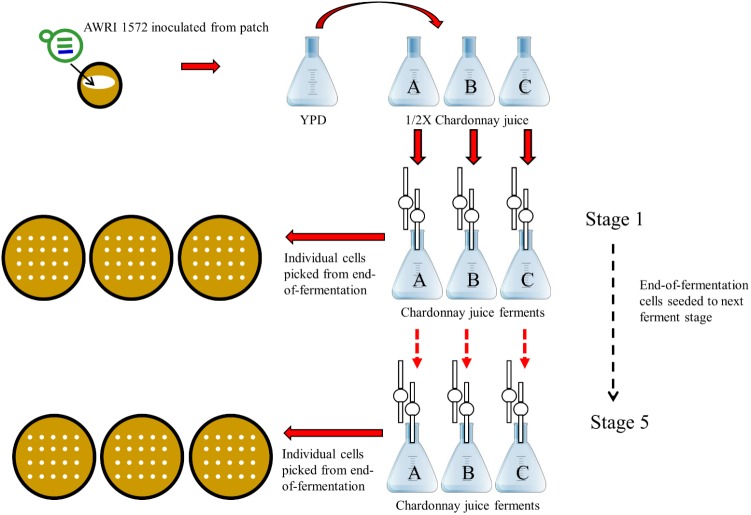
Design of adaptive evolution experiment to generate mutants with increased fitness in a winemaking context. Serial batch passaging of *S. cerevisiae* × *S. uvarum* interspecific hybrid AWRI1572 in Chardonnay juice.

Selection was performed in filter-sterilized Chardonnay juice fermentations (juice sourced from a vineyard in Blewitt Springs, SA, Australia): total sugars (glucose and fructose) 225 g/L, yeast assimilable nitrogen 226 mg/L, pH 3.39. This juice was supplemented with the 300 mg/L di-ammonium phosphate. Yeast strain AWRI 1572 was pre-cultured in YEPD medium (1% w/v yeast extract, 2% w/v peptone, 2% w/v glucose) for 2 days with an incubation temperature of 22°C and agitation rate of 150 rpm. The cells were then acclimatized to the higher sugar concentration of grape juice by 2 days of growth in ½ X Chardonnay juice (diluted with sterile water) before being inoculated from a cell density of 2 × 10^8^ cells per ml into 100 ml of Chardonnay juice to a final cell density of 2 × 10^6^ cells per ml. Triplicate fermentations were carried out under conditions described previously (Bellon et al., 2013). At completion of fermentation (when utilization of sugars cease) cells were isolated and 100 ml of fresh Chardonnay juice was then inoculated with 1 ml of 2 × 10^8^ cells per ml from the previous ferment to reach a cell density of 2 × 10^6^ cells per ml. This serial transfer procedure was repeated a further three times until five batch fermentations were completed (**Figure 1**).

### Chromosomal Analysis of Isolates From Passaged Populations

At fermentation completion of each passage a Singer^®^ micromanipulator was used to pick 20 individual cells from each replicate fermentation culture onto YEPD plates (60 individual cells in total from each passage), which were then incubated at 22°C until clonal colonies had formed. PCR-RFLP analysis on genomic DNA prepared from clonal colonies was performed using species-specific markers targeting each arm of the 16 *Saccharomyces* spp. chromosomes as previously described (Bellon et al., 2015) with additional markers for Chromosome 14 (**Table 1**) using *S. cerevisiae* S288C and *S. uvarum* (formerly *bayanus*) MCYC 623 sequences. Primer locations for each set of primers were assessed against work reported on *S. cerevisiae* and *S. uvarum* genomes (Fischer et al., 2000; Borneman et al., 2016) to identify markers affected by translocations. Chromosomes 1, 3, 5, 6, 9, 10, 11, 12, 13, and 14 are co-linear, while reciprocal translocations have occurred between 2/4, 7/16, and 8/15 chromosomes.

**Table 1:** Loci amplified, primer sequences and Restriction Enzymes for species-specific chromosomal markers.

Primer	Amplified loci in *S. cerevisiae*	Sequence	Restriction enzyme
1L	*OAF1* 48685–49530 bp	AGCACTCAAGCACATCGCCT	*Taqa*I
		AATATTCGCCACCTTGAGGG	
1R	*SWH1* 192768–193723 bp	AGTGCTCCATCTCATGCTCCA	*Mse*I
		TATTTGTCTCGATGGGGTGGT	
2L	*CDC27* 67260–68152 bp	GCATCTTTTTTCCTCCCAACT	*Taqa*I
		ACGCTGCCTGAAATCATGTAT	
2R	*CHS2* 312499–313610 bp	AACCATCCAACAAGACAGCA	*TaqaI*
		GCGACCAATTCCCAACAAA	
3L	*FUS1* 72313–73191 bp	ACCGCAGCATATACTGACACC	*TaqaI*
		ACTTTTTCACCCAGCGAGAT	
3R	*AGT15* 237694–238530 bp	CGCCATGTGGATAGATGATGA	*TaqaI*
		TGTGGATTCTGTGGTTGAACA	
4L	*UGA3* 156723–157490 bp	CGCCCATGAACCAGAACTACT	*Rsa*I
		GCCATAAGCGAAGGTTGTAA	
4R	*SNF1* 1412947–1413758 bp	GATTGCCGATTTTGGTTTGTC	*Mse*I
		TGATCCATGAAGGGTGATTG	
5L	*AFG1* 57189–58004 bp	TTTCAAGTCACTGACGTGGCA	*Rsa*I
		CATCTGCGATTTCTTGGCAA	
5R	*BCK2* 519304–520266 bp	TAGAAAACGAGCCAACACTGG	*Rsa*I
		CTCAATCCCAATCCCGTATT	
6L	*STE2* 82779–83727 bp	TTGTCATGTGGATGACATCGA	*Hae*III
		GGTGTGGGCAACTGATAAAA	
6R	*MET10* 214927–215981 bp	CCGTATCTGGAAAAGCATTG	*Taqa*I
		TAACTGGTTGCTTTGGAGATG	
7L	*GUS1* 3960–40528 bp	TCGTTTCCCACCTGAACCTT	*Taqa*I
		AAAGCCCAGATCAAGTTCCA	
7R	*GND2* 1005189–1006087 bp	GGTGATATGCAGTTGATTTGC	*Hae*III
		GATATATTACCTCCGTGCCCA	
8L	*OCA5* 46666–47424 bp	CGCCCTCTATCTTGTCTTTGT	*Taqa*I
		TGCCATCGTGAAATTTCTGC	
8R	*GND1* 471369–472285 bp	ATCTTTGATGCCAGGTGGTT	*Rsa*I
		TTGGCTGGCAATCTTTCAGA	
9L	*SUC2* 37698–38383 bp	TACAACAACACGAGTGGGTTT	*Rsa*I
		GAAAACTTGCGGACCAAAGA	
9R	*DAL4* 408763–409845 bp	CAGAGACTTGAAACCGGTTGA	*Taqa*I
		ATACATAGAGCCATTGCCACA	
10L	*ECM25* 54547–55202 bp	ATGAAATTGCCACAGGCAC	*Rsa*I
		TCATCAACAATTGGTAACGGA	
10R	*PMT4* 698900–699726 bp	CAACTGTAAGGTTCAGAGGCA	*Hae*III
		TTCTGGGTTCATTTCACCGT	
11L	*UBA1* 39239–40239 bp	TGGGTAAGGAAGCAATGTTGA	*Hae*III
		CACCTCTTGCCTGATAGGAAA	
11R	*PTR2* 616024–617343 bp	TCCGCACCATTCCAAAACTA	*Hae*III
		GCCAAACCAGTGAATAACCA	
12L	*FRA1* 82096–82864 bp	GAAGCTTTGGAAATGGCCAA	*Hae*III
		TGTGTGCGTTTTTTATTTCGA	
12R	*LEU3* 1037035–1037930 bp	TTAAGCGCCGACACTTCGT	*Mse*I
		CCATATGCTTCGCATTATTCC	
13L	*BUL2* 47651–48521 bp	CATCAATACCTCATGAGCGTC	*Taqa*I
		CGTCCAAAGTCCCGCTTATAT	
13R	*TDA1* 852727–853919 bp	ACCACAACTCCTTGGGCGAT	*Hae*III
		TCAACGTAAAGGTCAGGCAA	
14L	*LEM3* 32074–33077 bp	AGCCTGTGCGTACAAAGAACA	*Hae*III
		AATGGATTTCTACCGCCAA	
14LM	*CBK1* 333495–334599 bp	CGCCATTGAAAGAAATGAAAG	*Hae*III
		TTCATCTGCACCACCATGTCT	
14LC	*NOP2* 510540–511741 bp	TCATAAGAACAAGCAAGCCG	*Taqa*I
		TGTTGGTACAGCCTAGACGGT	
14RC	*LRO1* 640952–641994 bp	AAAGCTGGGGAGTTATTGGA	*Rsa*I
		TGGGTTGTTCACCCCGTATAT	
14R	*PPG1* 686010–687116 bp	TGGACGAATGTTTAGAAAGGC	*Hae*III
		TTAGAAGCAGATCTGGCTTGG	
15L	*GRE2* 43709–44440 bp	GTTCATTGCCCAACACATTG	*Hae*III
		AGCCTTTGCAACATCACGAA	
15R	*RDR1* 1051369–1052163 bp	GGCAAATCTCCATGTGAAATG	*Hae*III
		AATCTCATGATGCAGGCCAA	
16L	*SAM3* 23260–24344 bp	CCGCTTTGCTAATCGGTTTT	*Hae*III
		TCCTTGAGCTTTCAAAGCCA	
16R	*PRP4* 892389–893300 bp	ACAAAATGAAAGCACCGCTGA	*Hae*III
		CAAACAAGAGATCCATCGCA	

### Ploidy Determination Using Fluorescence Flow Cytometry Analysis

Colonies from YEPD plates were inoculated into liquid YEPD medium. Cells were harvested after 5 days of growth at 22°C and prepared in triplicate using a propidium iodide staining protocol for FACs analysis as described in Bellon et al. (2013). Cells were analyzed using a Guava^®^ easyCyte 12HT Sampling Flow Cytometer (Merck, Germany) instrument equipped with a 150 milliwatt DPSS laser emitting at 488 nm. Cells were detected at 583/26 nm using a Yellow B PMT filter with a flow rate of 7 μl/min and fluorescently plotted to a linear scale. Five thousand cells per sample were analyzed to obtain cell DNA intensities. Analysis was undertaken on six biological replicates of control ploidy strains and ancestral hybrid AWRI 1572 and 60 isolates from each fermentation series. Duplicate fluorescent readings were taken of all samples.

### Evaluation of Yeast Performance

Fermentations were conducted in two Chardonnay juices: the original Chardonnay juice containing 225 g/L reducing sugars, and the same juice supplemented with an addition of glucose and fructose, increasing the total reducing sugars concentration to 350 g/L. Fermentations were carried out in triplicate under conditions previously described (Bellon et al., 2015) with a single pre-conditioning step of growth in ½ X grape juice implemented for the 225 g/L sugar ferments, while an addition growth step in 1X chardonnay juice was used to pre-condition cells before inoculation into the high-sugar Chardonnay juice. Fermentation rates were determined by weight loss (OHRUS Adventurer^TM^ weighing meter) as a measure of CO_2_ egress from fermentation vessels.

Ethanol tolerance of yeast strains was determined by plating onto YEPD agar medium containing a range of ethanol concentrations (12, 14, and 16%). Plates were prepared when YEPD (plus 2% agar) medium was cooled to 50°C by the addition of a requisite volume of absolute ethanol. Strains were pre-cultured to a cell density of 2 × 10^8^ cells per ml in liquid YEPD for 2 days at 22°C and 5 μl of 10-fold serial dilutions spotted to plates and then further incubated at 22°C until sufficient colony growth could be observed.

### Analysis of Wines

Wines were analyzed using HPLC to determine concentrations of organic acids (malic, succinic, and acetic acids), residual sugars, glycerol, and ethanol as described previously (Bellon et al., 2013).

### Statistical Analyses

A Student’s *t*-test (*p* < 0.5) was used to determine differences between wines produced by different yeast strains.

## Results

### Chromosomal Analysis of Isolates From Populations of Passaged Yeast Hybrid AWRI 1572

**FIGURE 2:**
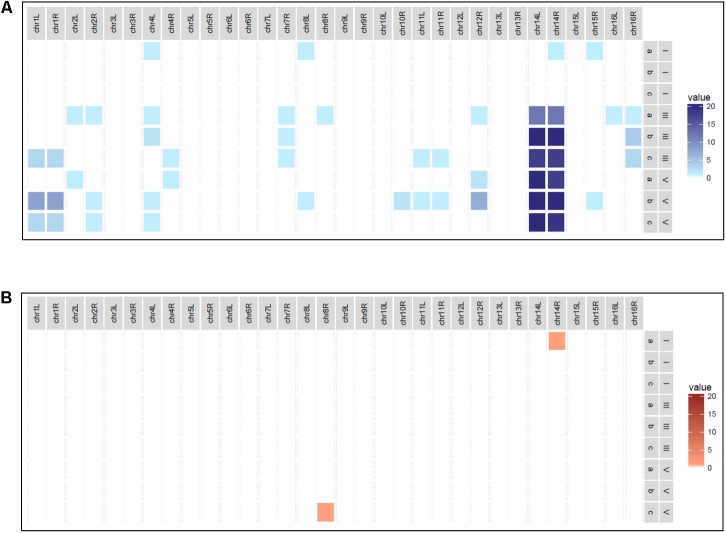
Heat map depicting AWRI1572 chromosomal loss in adaptive evolution experiment. **(A)** Blue box displays *S. uvarum* chromosomal loss with darkness of hue linked to increased frequency. **(B)** Red box displays *S. cerevisiae* chromosomal loss with darkness of hue linked to increased frequency. Scale 0–20 reflects loss per 20 isolates screened from triplicate ferments designated a, b, and c. Roman symbols I, III, and V refer to fermentation Stages 1, 3, and 5, respectively.

In order to generate an evolved hybrid strain with increased fitness in a fermentation context, consecutive grape juice batch fermentations were undertaken. Triplicate fermentations of Chardonnay grape juice inoculated with AWRI 1572 were serially passaged four times with cells harvested from each end-of-fermentation seeded into the next ferment at a rate of 2 × 10^6^ cells/ml (**Figure 1**). At the end of each fermentation single cells were harvested. Chromosomal compositions determined by PCR-RFLP revealed that loss of *S. uvarum* Chromosome 14 was the overriding chromosomal alteration occurring during the fermentation series (**Figure 2**).

Isolates harvested from the first stage ferment showed a stable genome with sporadic chromosomal loss detected in only four of the 60 isolates; a single marker in three isolates and two markers in the 4^th^ isolate (Supplementary Figure S1a). Whilst sporadic loss of markers continued to occur in the Stages 3 and 5 fermentations with some increases in frequency in Chromosomes 1, 12 (right arm only) and 16 (right arm only) (Supplementary Figures S1b,c), these alterations were either not present in the following fermentation stages (16R) or not identified in all triplicate ferments.

**FIGURE 3:**
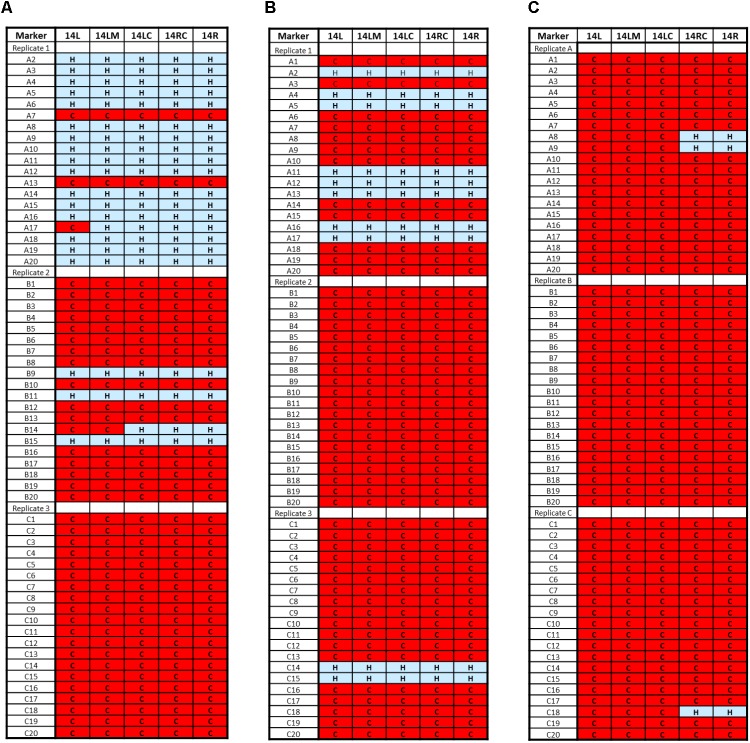
*Saccharomyces uvarum* Chromosome 14 loss from AWRI1572 during adaptive evolution experiment. Red ‘C’ box depicts only *S. cerevisiae* chromosome retained. Blue ‘H’ box depicts both *S. cerevisiae* and *S. uvarum* chromosome retained. **(A)** Stage 2 fermentation; **(B)** Stage 3 fermentation; **(C)** Stage 5 fermentation.

Analysis of cells harvested from Stage 2 fermentation series revealed a level of *S. uvarum* Chromosome 14 instability with whole or partial loss in some isolates from all replicate ferments (3 of 20 from replicate A, 16 of 20 from Replicate B, and all 20 isolates from Replicate C) (**Figure 3A**). Additional markers for Chromosome 14; 14ML (midway along the left arm), 14LC (proximal to the centromere on the left arm), and 14RC (proximal to the centromere on the right arm) (**Table 1**) were designed and used to confirm loss along the entire *S. uvarum* chromosome. The frequency of loss continued to increase in the Stage 3 ferments with 12 isolates of 20 from Replicate A, all 20 from Replicate B, and 18 of 20 from Replicate C having lost the complete *S. uvarum* Chromosome 14 (**Figure 3B**), culminating at Stage 5 with complete *S. uvarum* Chromosome 14 loss in 57 isolates while the remaining three isolates (two from Replicate A and one from Replicate C) showed partial loss, retaining the right arm (**Figure 3C**).

### Ploidy Determination of AWRI 1572 Cells Undergoing Serial Chardonnay Fermentations Using Fluorescence Flow Cytometry Analysis

Fluorescence flow cytometry analysis was used to confirm that fitness improvements of hybrid isolates were not due to a major change in ploidy. While this analysis is not sufficiently sensitive to distinguish differences in DNA content arising from loss of a single chromosome in a triploid background, it can be used to determine larger scale changes.

**FIGURE 4:**
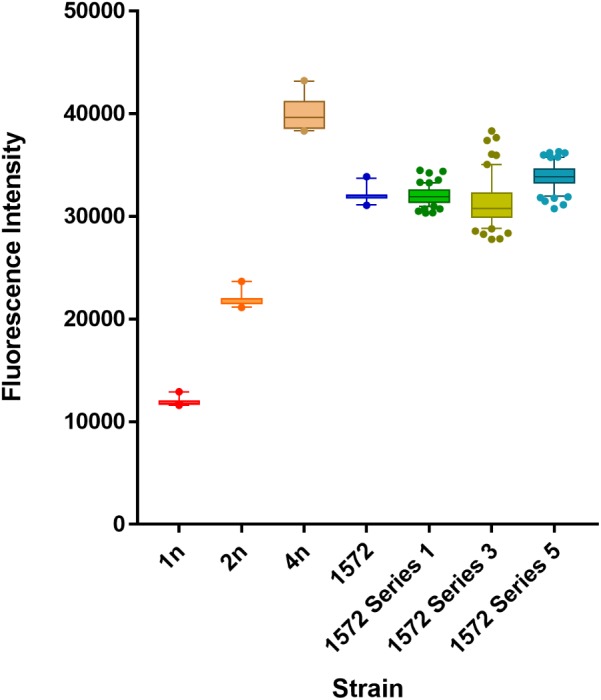
Ploidy levels of AWRI1572 during adaptive evolution experiment. Analyses of 60 isolates from each ferment series with mean ploidy values and whiskers at 10–90 percentile.

Fluorescence peak intensities for non-dividing G_0_ peaks showed diploid and tetraploid strain fluorescent levels approximately double or quadruple that of the control haploid strain respectively, while hybrid strain AWRI 1572 gave a G_0_ fluorescent peak level midway between diploid and tetraploid intensities. Fluorescence cell flow cytometry showed no discernible difference in ploidy status between the triploid ancestral hybrid strain AWRI 1572 and isolates (60 in total) from the Stage 1 ferment (**Figure 4**) and while isolates from Stage 3 and Stage 5 ferments showed greater diversity of DNA intensities, no isolates showed a gain or loss of complete ploidy level.

### Comparison of AWRI 1572 Fermentation Rates and Products vs. Evolved Hybrid Strain

Fermentations in Chardonnay juice were undertaken to establish that the loss of *S. uvarum* Chromosome 14 in the evolved hybrid strain had not compromised fermentation proficiency. One isolate, AWRI 2530, was chosen from Stage 3 that showed only loss of *S. uvarum* Chromosome 14 with an apparent 3n ploidy equivalent to the original hybrid strain.

**FIGURE 5:**
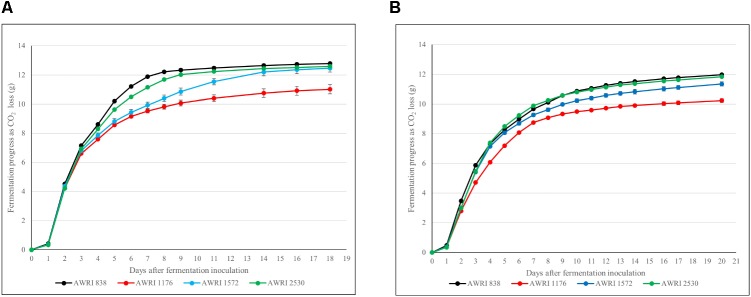
Chardonnay juice fermentation progression as determined by weight loss in grams of CO_2_. Data points are represented with standard deviation error bars: **(A)** 225 g/L sugar Chardonnay juice; **(B)** 350 g/L sugar Chardonnay juice.

The fermentation properties of parent strains of original hybrid (*S. cerevisiae* AWRI 838, *S. uvarum* AWRI 1176) and hybrid strains (ancestral AWRI 1572 and evolved AWRI 2530) were evaluated in two juices: the original Chardonnay juice containing 225 g/L reducing sugars, and a high-sugar juice fermentation with an addition of glucose and fructose to the Chardonnay juice increasing the total reducing sugars concentration to 350 g/L. The evolved hybrid strain (AWRI 2530) fermented at much faster rate than the original hybrid strain (AWRI 1572) in both fermentations with only a slightly reduced rate relative to the *S. cerevisiae* wine yeast parent strain, AWRI 838, in the 225 g/L sugar juice (**Figure 5A**) while matching the fermentation kinetics of the *S. cerevisiae* parent strain in the high-sugar ferment (**Figure 5B**). Isolates with only *S. uvarum* Chromosome 14 loss from each independently-evolving adaptive evolution replicate have been tested for fermentation efficiency. All of these isolates performed similarly (Supplementary Figure S5). The *S. uvarum* parent strain (AWRI 1176) showed the slowest fermentation rate in both fermentations and chemical analysis of the final wines established that this yeast was unable to complete fermentation in the 225 g/L sugar Chardonnay juice with a residual fructose concentration of 30g/L fermentation. Similar residual sugar levels were observed for the *S. cerevisiae* wine yeast parent strain and both hybrid strains in the high-sugar (350 g/L) ferments, but again, the *S. uvarum* parent strain was unable to utilize sugars to the same degree. Importantly, chemical analysis of the wines confirmed that the evolved strain AWRI 2530 had retained the desirable low-acetic acid production phenotype for which the ancestral hybrid strain was generated (Supplementary Table S1). No difference in organic acids, glycerol and ethanol concentrations was discernible between wines made by the evolved hybrid strain relative to the ancestral hybrid strain.

### Ethanol Tolerance

**FIGURE 6:**
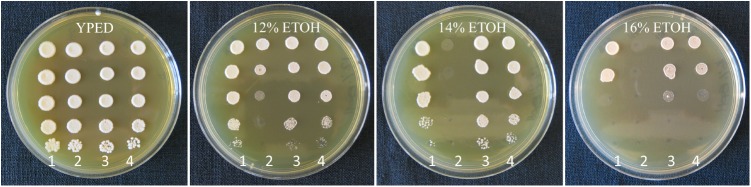
Ethanol tolerance assay plates. Plates left to right; YEPD, YEPD with 12% ethanol, 14% ethanol, or 16% ethanol. Strains were plated in columns at 10-fold serial dilutions from top to bottom; column1 AWRI838 (*S. cerevisiae*), column 2 AWRI1176 (*S. uvarum*), column 3 AWRI1572 (ancestral hybrid), column 4 AWRI2530 (evolved hybrid).

To verify that the important fermentation trait of high ethanol tolerance was retained in the evolved hybrid strain, growth on high ethanol medium was performed. Assay plating confirmed that the evolved strain AWRI 2530 retained the high ethanol tolerance trait of ancestral hybrid strain AWRI 1572, with tolerance slightly higher than the original *S. cerevisiae* grandparent wine yeast strain AWRI 838, visualized as denser cell growth at 14 and 16% ethanol (**Figure 6**).

### Competitive Growth Between Parent Hybrid (AWRI 1572) and the Evolved Isolate (AWRI 2530)

A competitive growth assay was carried out in Chardonnay juice to validate the improved fitness status of the evolved isolate relative to the ancestral hybrid strain in a fermentation context.

A co-fermentation of AWRI 1572 and AWRI 2530 was conducted in triplicate using Chardonnay juice. Both strains were subcultured individually in YEPD for 2 days and then acclimatized to Chardonnay grape juice by growth in 1/2X juice for 2 days. Equal numbers of cells (1 × 10^6^ cells/ml) of AWRI 1572 and AWRI 2530 were then co-inoculated into triplicate, full strength Chardonnay juice. At fermentation completion, cells were then passaged into a second stage ferment at 2 × 10^6^ cells/ml.

Cells were harvested at end-of-fermentation from the first ferment, and early stationary in the Stage 2 ferment (day 3). One hundred cells from each fermentation medium were picked to YPD plates using a Singer manipulator.

**FIGURE 7:**
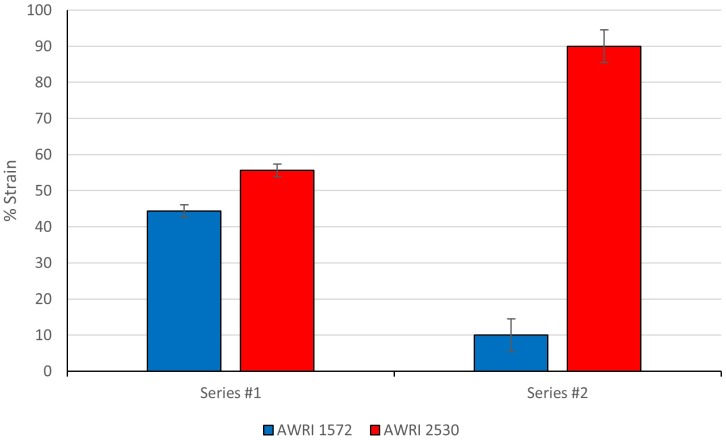
Competition assay of AWRI1572 vs AWRI2530. Data points are represented with error bars showing 95% Confidence Intervals. 100 isolates analyzed for presence/absence of *S. uvarum* Chromosome 14 from each triplicate fermentation. Student’s *t*-test: Series #1 8.13E-04, Series #2 1.64E-05.

Colonies were identified through PCR-RFLP species specific marker (14L) using primers targeting the left arm of Chromosome 14 which was missing in the evolved hybrid (AWRI 2530). Analysis of cells harvested from the final stage of the competition fermentation revealed that AWRI 2530 had out-competed the ancestral strain and cell numbers had risen to 90% of the population (**Figure 7**) with replicate ferments having AWRI 2530 populations ranging from 86 to 94%. However, only a slight growth advantage was observed during first ferment as AWRI 2530 cell numbers increased to a final proportion of only 55% from the initial inoculum of 50%.

Twenty AWRI 2530-identified cells from each Stage 2 replicate fermentation were analyzed using the 32 PCR-RFLP chromosomal marker system to confirm no further genomic instability in the evolved strain. Of the 60 clones evaluated only two showed any chromosomal mutations; both were lacking *S. uvarum* Chromosome 4L marker (Supplementary Figure S2) while Fluorescence flow cytometry confirmed that the overall DNA content of this strain remained stable (Supplementary Figure S3).

In order to establish that no loss of *S. uvarum* chromosome 14 occurred in the parent hybrid strain (AWRI 1572) during the experiment, separate ferments where undertaken in parallel in which this strain was the sole yeast inoculated into the grape must. PCR-RFLP analysis of 100 clones from each triplicate AWRI 1572-solo ferment confirmed that the ancestral strain AWRI 1572 was stable with respect to *S. uvarum* Chromosome 14 in the context of these two sequential Chardonnay juice fermentations (Supplementary Figure S4).

## Discussion

The research described in the current manuscript builds upon work previously reported from our laboratory on the design and generation of *S. cerevisiae* × *S. uvarum* interspecific yeast hybrids targeted to the phenotype of reduced production of acetic acid in grape juice fermentation. Here, we describe the selection of an improved hybrid with increased fitness in grape juice fermentation using serial, grape juice fermentations as summarized in **Figure 1**. Identification of a mutant with an improved phenotype was made possible by the introduction of a novel approach to screening for candidate strains with increased fitness from evolving populations: a set 32 PCR/RFLP primer and restriction enzyme pairs designed to target each arm of the 16 *Saccharomyces* chromosomes was used to confirm presence/absence of *S. cerevisiae* and *S. uvarum* chromosomes in cells isolated from different stages of the evolving population. This enabled retrospective identification of cells that had the most represented endpoint chromosomal complement, but from an early timepoint.

Traditional approaches to adaptive evolution in yeast strains utilize end-point sampling from multi-batch culture growths (McBryde et al., 2006; Cadière et al., 2011), or steady state growth conditions using chemostats with population sampling following 100s of cell generations (Hansche et al., 1978; Gresham et al., 2008; Kvitek and Sherlock, 2011; Kutyna et al., 2012). This carries the risk of selecting for mutations that shape phenotypes over and above that which is targeted. Identifying a mutant with the desired phenotype from an early timepoint in the evolutionary process would reduce this risk.

Hybrid isolates recovered from the final (5^th^) round ferment revealed that chromosomal mutations in *S. uvarum* Chromosome 14 occurred in all isolates analyzed: 95% of isolates lost the entire chromosome while 5% still retained the right arm.

While minor Chromosome 14 instability was identified in isolates from the first stage fermentation, by the completion of the second fermentation stage it was evident that *S. uvarum* Chromosome 14 was preferentially lost (67% of isolates showed partial or whole chromosome loss) and the frequency of this karyotype increased to 100% over subsequent passaging steps. A possible reason for this was that loss of *S. uvarum* Chromosome 14 leads to increased fitness in this interspecific hybrid.

Loss of other chromosomal markers during later stages of the fermentation series was also identified (10–15% of clones showed an alteration in Chromosomes 1, 12, or 16 during Stages 3–5) but none of these showed a frequency increase in all replicates over the course of the experiment. While some of the single arm marker loss identified in *S. uvarum* chromosomes containing translocations can be attributed to marker location (e.g., 2L pairs with 4R while 2R pairs with 4L) a number of unmatched losses were detected in marker 16R from the Stage 3 ferment. Interestingly, no loss of marker 16R was identified in cells harvested from the final stage of adaptive evolution and perhaps loss of the right arm of *S. uvarum* Chromosome 16 resulted in decreased fitness relative to the rest of the population. Given that the frequency of *S. uvarum* Chromosome 14 loss was 100% by the end of the adaptive evolution series (95% showing loss of the entire chromosome), and that an evolved strain with only loss of *S. uvarum* Chromosome 14 identified displayed increased fitness relative to the ancestral hybrid strain, it is unlikely that these other changes, at least in the genetic background of AWRI 1572, and in Chardonnay juice led to increased fitness.

An isolate (AWRI 2530) with loss of *S. uvarum* Chromosome 14 but no other detectable changes in karyotype was chosen for further characterization. A competitive growth assay was carried out in Chardonnay juice to validate the improved fitness status of the evolved isolate relative to the ancestral hybrid strain in a fermentation context. As the first evidence of *S. uvarum* chromosome 14 genomic instability in the adaptive evolution fermentation experiment occurred in isolates harvested from the end-of-fermentation Stage 2 culture, sampling of the 2^nd^ stage competition ferment was undertaken very early in the fermentation (at day 3: it took 14 days for the fermentation to reach completion). Separate ferments with ancestral parent hybrid AWRI 1572 alone were used to establish that Chromosome 14 instability was not an issue for the duration of the competition experiment. Serial growth competition in Chardonnay juice between the original hybrid (AWRI 1572) and the above isolate demonstrated that loss of *S. uvarum* Chromosome 14 is likely to contribute to the observed increase in fitness, but we cannot exclude the possibility that other mutations contributed to this phenotype.

Chromosomal assessment of AWRI 2530 isolates from the competition assay showed a stable hybrid karyotype. Whilst some slight differences in DNA fluorescence levels were detected during the two-series fermentation, no overall change in ploidy was observed; all isolates seemingly retained their triploid status (although differences in chromosomal aneuploidy levels cannot be discerned by our methods). Interestingly, other studies describing the stabilization of synthetic polyploid interspecific hybrids have reported ploidy stabilization to triploid levels; a tetraploid *S. cerevisiae* × *S. kudriavzevii* hybrid showed a loss of DNA content, stabilizing at a level similar to triploid when undergoing fermentation stresses (Pérez-Través et al., 2014), while in another study diploid *S. cerevisae* × *S. bayanus* hybrids increased their DNA content by 60% (approximating triploid ploidy levels) after 50–80 vegetative generations (Kunicka-Styczyńska and Rajkowska, 2011).

Fermentation kinetics showed that the evolved strain had an increased fermentation performance relative to the original hybrid as it was able to metabolize sugars at a faster rate and complete fermentation in a shorter time-frame. Ethanol tolerance was not diminished as both hybrid strains displayed slightly higher tolerance than the wine yeast parent, which was evident at a concentration of 14% ethanol. Isolates with only *S. uvarum* Chromosome 14 loss from each independently-evolving adaptive evolution replicate have been tested for fermentation efficiency. All of these isolates performed similarly (Supplementary Figure S5).

Analysis of the resultant wines indicated that the desirable winemaking traits of the original hybrid AWRI 1572 had not been compromised in the evolved strain as no difference in secondary fermentation products and ethanol production was seen and the evolved strain retained the low volatile acidity production trait of the original hybrid strain.

Kingdoms that utilize a sexual cycle generate variability by recombination and chromosomal assortment. Interspecific hybridization (mating between closely related species) brings even greater novelty to an organism, enhancing genetic and biochemical flexibility relative to the parents. On the other hand, plant studies have shown that interaction between different genomes with inherent incompatibilities can lead to genomic instability with alterations such as chromosomal losses, translocations, gene repetitions, and silencing (Comai et al., 2000; Adams and Wendel, 2005). Studies of natural *Saccharomyces* interspecific hybrids have identified evidence of recombination between parental chromosomes and depleted DNA sequences at subtelomeric regions (Bond et al., 2004; Pfliegler et al., 2014), while chromosome aneuploidy and genome rearrangements were identified in laboratory-generated interspecific hybrids undergoing adaptive evolution over 100s of generations (Smukowski Heil et al., 2017). In addition, meiotic events have been shown to precipitate extensive genomic changes in laboratory generated *Saccharomyces* interspecific hybrids with elimination of uni-parental chromosomes (in particular non-*cerevisiae*) and genomic rearrangements (Antunovics et al., 2005; Pfliegler et al., 2014; Lopandic et al., 2016; Krogerus et al., 2017).

In addition, mating that results in polyploidy provides redundancy that can accelerate genomic change (Selmecki et al., 2015) and function divergence. Polyploidy has been a very important factor in plant evolution (Wendel, 2000) and many flowering plants and common crop plants (i.e., wheat, rice, coffee, and banana) have polyploid derivation. While the cereal species of wheat and rice are evolutionary hybrids and their genomic stabilization may have been the result of eons of minor genomic changes, studies of incipient *Brassica* interspecific hybrids have shown that rapid and extensive genomic changes can occur within five generations of hybridization and that a relationship exists between frequency of change and divergence of parental genomes (Song et al., 1995). It is important to note that genomic instability in interspecific plants is a feature of meiotic divisions as opposed to the mitotic growth in the adaptive evolution experiments described in the current manuscript. With this in mind (and while it is unlikely in the context of the work described in the current study) meiotic segregation cannot be excluded from having a role contributing to chromosomal loss in the evolved strain genome.

Genome stability and the maintenance of appropriate gene regulation is essential for normal functioning and cell viability. However, a certain amount of genome plasticity can be an advantage when organisms encounter challenging environs, potentially enabling acclimatization to changing conditions. A model, ‘fast adaptive genome evolution’ (FAGE), has been proposed to explain the roles of changing clonal population during yeast fermentation (Sipiczki, 2011). This model involves sporulation at the end of fermentation, followed by either autodiploidization in the next vintage or conjugation of non-sister spore clones. Whilst yeast meiosis and sporulation can occur in fermentation environs when nutrients become depleted, the adaptive evolution experiment in the current study involved microscopic inspection of end-of-ferment culture at the time of yeast cell isolation to safeguard that only mitotic growth had contributed to genomic changes in the evolving population.

Genome instability on Chromosome 14 in *S. cerevisiae* × *S. uvarum* diploid hybrids exposed to nitrogen limiting conditions has been reported earlier by Dunn et al. (2013). In their study, reciprocal translocations between *MEP2* (a high-affinity ammonium permease) occurred, resulted in chimeric chromosomes each carrying a MEP2 fusion gene. This chromosomal rearrangement also increased hybrid fitness, allowing evolved hybrid strains to grow faster under nitrogen-limitation than ancestral hybrids. An evolutionary study involving a different alloploid interspecific hybrid strain, *Saccharomyces pastorianus*, also showed instability in Chromosome 14 with loss of *S. cerevisiae* right arm copy number (Brickwedde et al., 2017).

Recently, genomic sequencing of a small number of natural wine yeast hybrids was reported (Borneman et al., 2016) and, while the only *S. cerevisiae* × *S. uvarum* hybrid sequenced (Lalvin S6U) had retained the *S. uvarum* Chromosome 14, two *S. cerevisiae* × *S. kudriavzevii* natural wine yeast hybrids (Enoferm Assmunshansen and Maurivin EP2) lost the non- *S. cerevisiae* Chromosome 14 (however, both these hybrids also sustained large losses of *S. kudriavzevii* genome and have *S. kudriavzevii* contributions from only six chromosomes).

In the current study, little evidence of partial loss of *S. uvarum* Chromosome 14 was identified by the chromosomal marker system used, with only six isolates from a total of 240 revealing partial alteration. This could mean that partial loss of Chromosome 14 is rare, fundamentally unstable, or leads to decreased fitness.

The cause of increased fitness in the evolved strain remains to be determined. Loss of the *S. uvarum* Chromosome 14 may, for example, have impacted on acclimatization to fresh medium leading to a decreased lag phase, faster growth in exponential phase, increased cell population at stationary phase, the ability to tolerate stresses such high sugar and high ethanol concentrations or the ability to uptake and metabolize sugars at a faster rate. While there is high DNA sequence divergence between *S. cerevisiae* and *S. uvarum* [similar to that between human and mouse with 62% nucleotide identity in aligned positions (Kellis et al., 2003)] *S. cerevisiae* and *S. uvarum* Chromosomes 14 are co-linear with no translocations reported (Fischer et al., 2000) and there is potential for a number of genes on this chromosome to impact on cell fitness in a fermentation context: roles in stress tolerance, e.g., *FIG4*, *WSC2*, *HCH1*, *SKO1*, *CRZ1*, and *PDR18*; roles in glucose metabolism, e.g., *HXT14*, *GCR2*, *YCK2*, and *SSN8*; roles in cell growth, e.g., *IES2* and *YGP1*.

## Conclusion

We report the successful generation of an evolved interspecific wine yeast hybrid with increased fitness in a fermentation context relative to the ancestral hybrid strain. This was achieved using a novel screening approach that utilized chromosomal mutations as markers for the trait of interest. The evolved hybrid strain retained the targeted fermentation trait of reduced volatile acidity production while exhibiting improved fermentation kinetics. The chromosomal marker system employed allowed the pattern of genomic plasticity that arose during the evolution of the interspecific hybrid to be exposed and future work on individual isolates from the evolution study may reveal information about genomic alterations that lead to interspecific yeast hybrid stabilization.

## Author Contributions

JB designed and undertook all the experimental work, interpreted the data, and wrote the manuscript. CF, AB, and PC assisted in experimental design, supervised experimental work, helped to evaluate, and edit the manuscript.

## Conflict of Interest Statement

The authors declare that the research was conducted in the absence of any commercial or financial relationships that could be construed as a potential conflict of interest.
