# Supplementary material for: A Novel Approach to Isolating Improved Industrial Interspecific Wine Yeasts Using Chromosomal Mutations as Potential Markers for Increased Fitness
Source: Front Microbiol. 2018 Jul 3;9:1442. doi: 10.3389/fmicb.2018.01442 (PMC6043810; doi:10.3389/fmicb.2018.01442)
Supplement: Supplementary file 1 [file Data_Sheet_1.pdf]

frontiers

Supplementary Material

A novel approach to isolating improved industrial interspecific wine yeasts using chromosomal mutations as a marker for increased fitness.

Jennifer R Bellon*, Anthony R Borneman, Christopher M Ford, Paul J Chambers

*** Correspondence:** Corresponding Author: jenny.bellon@awri.com.au

Supplementary Figure S1. Chromosomal marker loss from AWRI 1572 following Stages 1, 3 and 5 of the adaptive evolution experiment.
Red ‘C’ box depicts S. cerevisiae marker only retained, yellow ‘U’ box depicts *S. uvarum* marker only retained, blue ‘H’ box depicts both *S. cerevisiae* and *S. uvarum* markers retained. Chromosomal markers are shown along the top horizontal of the figure and isolates are numbered 1-20 in triplicates A, B & C along the left vertical. **Figure S1a. Stage 1; Figure S1b. Stage 3; Figure S1c. Stage 5**

Supplementary Figure S2. Chromosomal marker 4L loss from AWRI 2530 isolates following Stage 2 competition experiment.
Left gel ‘A’ replicate AWRI 2530 isolates and right gel ‘B’ replicate AWRI 2530 isolates with 4L marker (top) and 4R marker (bottom). In each gel; lane 1 100bp ladder, lane 2 AWRI 838, lane 3 AWRI 1176, lane 4 AWRI 2530, lanes 5 to 24 isolates of AWRI 2530. White arrows point to marker loss.

Supplementary Figure S3. Ploidy levels of AWRI 2530 following Stage 2 competition experiment.
Analyses of 60 isolates from each Stage 2 replicate ferment with mean ploidy values and whiskers at 10-90 percentile.

Supplementary Figure S4. PCR-RFLP 14L chromosomal marker from ancestral hybrid AWRI 1572 isolates following Stage 2 competition fermentation.
1st row Replicate ‘A’ isolates 1-50, 2nd row Replicate ‘A’ isolates 51-100, 3rd row Replicate ‘B’ isolates 1-50, 4th row Replicate ‘B’ isolates 51-100, 5th row Replicate ‘C’ isolates 1-50, 6th row Replicate ‘C’ isolates 51-100. In each gel; lane 1 100bp ladder, lane 2 AWRI 838, lane 3 AWRI 1176, lane 4 AWRI 1572, lanes 5 to 54 hybrid isolates of AWRI 1572 following Stage 2 competition fermentation.

Supplementary Figure S5. Chardonnay juice (275 g/L sugar) fermentation progression of ancestral hybrid AWRI 1572 and evolved isolates with only *S. uvarum* Chromosome 14 loss. AWRI 2530, isolate from Replicate B Stage 3; AWRI 1448, isolate from Replicate A Stage 3; AWRI 1449, isolate from Replicate C Stage 3.
Data points are represented with standard deviation error bars.

Supplementary Data Table S1. Secondary fermentation products present in Chardonnay wine produced using ancestral hybrid AWRI 1572 and evolved isolate AWRI 2530. * % (v/v). Within a row, values connected by same letter are not significantly different (p<0.05)**.** All values in the table are means of triplicates with standard deviations

Supplementary Figure S1a.

Marker	1L	1R	2L	2R	3L	3R	4L	4R	5L	5R	6L	6R	7L	7R	8L	8R	9L	9R	10L	10R	11L	11R	12L	12R	13L	13R	14L	14R	15L	15R	16L	16R
Replicate A																																
A1	H	H	H	H	H	H	H	H	H	H	H	H	H	H	H	H	H	H	H	H	H	H	H	H	H	H	H	H	H	H	H	H
A2	H	H	H	H	H	H	H	H	H	H	H	H	H	H	H	H	H	H	H	H	H	H	H	H	H	H	H	H	H	H	H	H
A3	H	H	H	H	H	H	H	H	H	H	H	H	H	H	H	H	H	H	H	H	H	H	H	H	H	H	H	H	H	H	H	H
A4	H	H	H	H	H	H	H	H	H	H	H	H	H	H	H	H	H	H	H	H	H	H	H	H	H	H	H	U	H	H	H	H
A5	H	H	H	H	H	H	H	H	H	H	H	H	H	H	H	H	H	H	H	H	H	H	H	H	H	H	H	H	H	H	H	H
A6	H	H	H	H	H	H	H	H	H	H	H	H	H	H	H	H	H	H	H	H	H	H	H	H	H	H	H	H	H	H	H	H
A7	H	H	H	H	H	H	H	H	H	H	H	H	H	H	H	H	H	H	H	H	H	H	H	H	H	H	H	H	H	H	H	H
A8	H	H	H	H	H	H	H	H	H	H	H	H	H	H	H	H	H	H	H	H	H	H	H	H	H	H	H	H	H	H	H	H
A9	H	H	H	H	H	H	H	H	H	H	H	H	H	H	H	H	H	H	H	H	H	H	H	H	H	H	H	H	H	H	H	H
A10	H	H	H	H	H	H	C	H	H	H	H	H	H	H	H	H	H	H	H	H	H	H	H	H	H	H	H	H	H	H	H	H
A11	H	H	H	H	H	H	H	H	H	H	H	H	H	H	C	H	H	H	H	H	H	H	H	H	H	H	H	H	H	C	H	H
A12	H	H	H	H	H	H	H	H	H	H	H	H	H	H	H	H	H	H	H	H	H	H	H	H	H	H	H	C	H	H	H	H
A13	H	H	H	H	H	H	H	H	H	H	H	H	H	H	H	H	H	H	H	H	H	H	H	H	H	H	H	H	H	H	H	H
A14	H	H	H	H	H	H	H	H	H	H	H	H	H	H	H	H	H	H	H	H	H	H	H	H	H	H	H	H	H	H	H	H
A15	H	H	H	H	H	H	H	H	H	H	H	H	H	H	H	H	H	H	H	H	H	H	H	H	H	H	H	H	H	H	H	H
A16	H	H	H	H	H	H	H	H	H	H	H	H	H	H	H	H	H	H	H	H	H	H	H	H	H	H	H	H	H	H	H	H
A17	H	H	H	H	H	H	H	H	H	H	H	H	H	H	H	H	H	H	H	H	H	H	H	H	H	H	H	H	H	H	H	H
A18	H	H	H	H	H	H	H	H	H	H	H	H	H	H	H	H	H	H	H	H	H	H	H	H	H	H	H	H	H	H	H	H
A19	H	H	H	H	H	H	H	H	H	H	H	H	H	H	H	H	H	H	H	H	H	H	H	H	H	H	H	H	H	H	H	H
A20	H	H	H	H	H	H	H	H	H	H	H	H	H	H	H	H	H	H	H	H	H	H	H	H	H	H	H	H	H	H	H	H
Replicate B																																
B1	H	H	H	H	H	H	H	H	H	H	H	H	H	H	H	H	H	H	H	H	H	H	H	H	H	H	H	H	H	H	H	H
B2	H	H	H	H	H	H	H	H	H	H	H	H	H	H	H	H	H	H	H	H	H	H	H	H	H	H	H	H	H	H	H	H
B3	H	H	H	H	H	H	H	H	H	H	H	H	H	H	H	H	H	H	H	H	H	H	H	H	H	H	H	H	H	H	H	H
B4	H	H	H	H	H	H	H	H	H	H	H	H	H	H	H	H	H	H	H	H	H	H	H	H	H	H	H	H	H	H	H	H
B5	H	H	H	H	H	H	H	H	H	H	H	H	H	H	H	H	H	H	H	H	H	H	H	H	H	H	H	H	H	H	H	H
B6	H	H	H	H	H	H	H	H	H	H	H	H	H	H	H	H	H	H	H	H	H	H	H	H	H	H	H	H	H	H	H	H
B7	H	H	H	H	H	H	H	H	H	H	H	H	H	H	H	H	H	H	H	H	H	H	H	H	H	H	H	H	H	H	H	H
B8	H	H	H	H	H	H	H	H	H	H	H	H	H	H	H	H	H	H	H	H	H	H	H	H	H	H	H	H	H	H	H	H
B9	H	H	H	H	H	H	H	H	H	H	H	H	H	H	H	H	H	H	H	H	H	H	H	H	H	H	H	H	H	H	H	H
B10	H	H	H	H	H	H	H	H	H	H	H	H	H	H	H	H	H	H	H	H	H	H	H	H	H	H	H	H	H	H	H	H
B11	H	H	H	H	H	H	H	H	H	H	H	H	H	H	H	H	H	H	H	H	H	H	H	H	H	H	H	H	H	H	H	H
B12	H	H	H	H	H	H	H	H	H	H	H	H	H	H	H	H	H	H	H	H	H	H	H	H	H	H	H	H	H	H	H	H
B13	H	H	H	H	H	H	H	H	H	H	H	H	H	H	H	H	H	H	H	H	H	H	H	H	H	H	H	H	H	H	H	H
B14	H	H	H	H	H	H	H	H	H	H	H	H	H	H	H	H	H	H	H	H	H	H	H	H	H	H	H	H	H	H	H	H
B15	H	H	H	H	H	H	H	H	H	H	H	H	H	H	H	H	H	H	H	H	H	H	H	H	H	H	H	H	H	H	H	H
B16	H	H	H	H	H	H	H	H	H	H	H	H	H	H	H	H	H	H	H	H	H	H	H	H	H	H	H	H	H	H	H	H
B17	H	H	H	H	H	H	H	H	H	H	H	H	H	H	H	H	H	H	H	H	H	H	H	H	H	H	H	H	H	H	H	H
B18	H	H	H	H	H	H	H	H	H	H	H	H	H	H	H	H	H	H	H	H	H	H	H	H	H	H	H	H	H	H	H	H
B19	H	H	H	H	H	H	H	H	H	H	H	H	H	H	H	H	H	H	H	H	H	H	H	H	H	H	H	H	H	H	H	H
B20	H	H	H	H	H	H	H	H	H	H	H	H	H	H	H	H	H	H	H	H	H	H	H	H	H	H	H	H	H	H	H	H
Replicate C																																
C1	H	H	H	H	H	H	H	H	H	H	H	H	H	H	H	H	H	H	H	H	H	H	H	H	H	H	H	H	H	H	H	H
C2	H	H	H	H	H	H	H	H	H	H	H	H	H	H	H	H	H	H	H	H	H	H	H	H	H	H	H	H	H	H	H	H
C3	H	H	H	H	H	H	H	H	H	H	H	H	H	H	H	H	H	H	H	H	H	H	H	H	H	H	H	H	H	H	H	H
C4	H	H	H	H	H	H	H	H	H	H	H	H	H	H	H	H	H	H	H	H	H	H	H	H	H	H	H	H	H	H	H	H
C5	H	H	H	H	H	H	H	H	H	H	H	H	H	H	H	H	H	H	H	H	H	H	H	H	H	H	H	H	H	H	H	H
C6	H	H	H	H	H	H	H	H	H	H	H	H	H	H	H	H	H	H	H	H	H	H	H	H	H	H	H	H	H	H	H	H
C7	H	H	H	H	H	H	H	H	H	H	H	H	H	H	H	H	H	H	H	H	H	H	H	H	H	H	H	H	H	H	H	H
C8	H	H	H	H	H	H	H	H	H	H	H	H	H	H	H	H	H	H	H	H	H	H	H	H	H	H	H	H	H	H	H	H
C9	H	H	H	H	H	H	H	H	H	H	H	H	H	H	H	H	H	H	H	H	H	H	H	H	H	H	H	H	H	H	H	H
C10	H	H	H	H	H	H	H	H	H	H	H	H	H	H	H	H	H	H	H	H	H	H	H	H	H	H	H	H	H	H	H	H
C11	H	H	H	H	H	H	H	H	H	H	H	H	H	H	H	H	H	H	H	H	H	H	H	H	H	H	H	H	H	H	H	H
C12	H	H	H	H	H	H	H	H	H	H	H	H	H	H	H	H	H	H	H	H	H	H	H	H	H	H	H	H	H	H	H	H
C13	H	H	H	H	H	H	H	H	H	H	H	H	H	H	H	H	H	H	H	H	H	H	H	H	H	H	H	H	H	H	H	H
C14	H	H	H	H	H	H	H	H	H	H	H	H	H	H	H	H	H	H	H	H	H	H	H	H	H	H	H	H	H	H	H	H
C15	H	H	H	H	H	H	H	H	H	H	H	H	H	H	H	H	H	H	H	H	H	H	H	H	H	H	H	H	H	H	H	H
C16	H	H	H	H	H	H	H	H	H	H	H	H	H	H	H	H	H	H	H	H	H	H	H	H	H	H	H	H	H	H	H	H
C17	H	H	H	H	H	H	H	H	H	H	H	H	H	H	H	H	H	H	H	H	H	H	H	H	H	H	H	H	H	H	H	H
C18	H	H	H	H	H	H	H	H	H	H	H	H	H	H	H	H	H	H	H	H	H	H	H	H	H	H	H	H	H	H	H	H
C19	H	H	H	H	H	H	H	H	H	H	H	H	H	H	H	H	H	H	H	H	H	H	H	H	H	H	H	H	H	H	H	H
C20	H	H	H	H	H	H	H	H	H	H	H	H	H	H	H	H	H	H	H	H	H	H	H	H	H	H	H	H	H	H	H	H

Supplementary Figure S1b

Marker	1L	1R	2L	2R	3L	3R	4L	4R	5L	5R	6L	6R	7L	7R	8L	8R	9L	9R	10L	10R	11L	11R	12L	12R	13L	13R	14L	14LM	14LC	14RC	14R	15L	15R	16L	16R
Replicate A																																			
A1	H	H	H	H	H	H	H	H	H	H	H	H	H	H	H	H	H	H	H	H	H	H	H	H	H	H	C	C	C	C	C	H	H	H	H
A2	H	H	H	H	H	H	H	H	H	H	H	H	H	H	H	H	H	H	H	H	H	H	H	H	H	H	H	H	H	H	H	H	H	H	H
A3	H	H	H	H	H	H	H	H	H	H	H	H	H	H	H	C	H	H	H	H	H	H	H	H	H	H	C	C	C	C	C	H	H	H	H
A4	H	H	H	H	H	H	H	H	H	H	H	H	H	H	H	H	H	H	H	H	H	H	H	H	H	H	H	H	H	H	H	H	H	H	H
A5	H	H	H	H	H	H	H	H	H	H	H	H	H	H	H	H	H	H	H	H	H	H	H	H	H	H	H	H	H	H	H	H	H	H	H
A6	H	H	H	H	H	H	H	H	H	H	H	H	H	H	H	H	H	H	H	H	H	H	H	H	H	H	C	C	C	C	C	H	H	H	H
A7	H	H	H	H	H	H	C	H	H	H	H	H	H	H	H	H	H	H	H	H	H	H	H	H	H	H	C	C	C	C	C	H	H	H	H
A8	H	H	H	H	H	H	H	H	H	H	H	H	H	H	H	H	H	H	H	H	H	H	H	H	H	H	C	C	C	C	C	H	H	H	H
A9	H	H	H	H	H	H	H	H	H	H	H	H	H	H	H	H	H	H	H	H	H	H	H	H	H	H	C	C	C	C	C	H	H	H	H
A10	H	H	C	C	H	H	H	H	H	H	H	H	H	C	H	H	H	H	H	H	H	H	H	H	H	H	C	C	C	C	C	H	H	C	H
A11	H	H	H	H	H	H	H	H	H	H	H	H	H	H	H	H	H	H	H	H	H	H	H	H	H	H	H	H	H	H	H	H	H	H	H
A12	H	H	H	H	H	H	H	H	H	H	H	H	H	H	H	H	H	H	H	H	H	H	H	H	H	H	H	H	H	H	H	H	H	H	H
A13	H	H	H	H	H	H	H	H	H	H	H	H	H	H	H	H	H	H	H	H	H	H	H	H	H	H	H	H	H	H	H	H	H	H	C
A14	H	H	H	H	H	H	H	H	H	H	H	H	H	H	H	H	H	H	H	H	H	H	H	H	H	H	C	C	C	C	C	H	H	H	H
A15	H	H	H	H	H	H	H	H	H	H	H	H	H	H	H	H	H	H	H	H	H	H	H	H	H	H	C	C	C	C	C	H	H	H	H
A16	H	H	H	H	H	H	H	H	H	H	H	H	H	H	H	H	H	H	H	H	H	H	H	C	H	H	H	H	H	H	H	H	H	H	H
A17	H	H	H	H	H	H	H	H	H	H	H	H	H	H	H	H	H	H	H	H	H	H	H	H	H	H	H	H	H	H	H	H	H	H	H
A18	H	H	H	H	H	H	H	H	H	H	H	H	H	H	H	H	H	H	H	H	H	H	H	H	H	H	C	C	C	C	C	H	H	H	H
A19	H	H	H	H	H	H	H	H	H	H	H	H	H	H	H	H	H	H	H	H	H	H	H	H	H	H	C	C	C	C	C	H	H	H	H
A20	H	H	H	H	H	H	H	H	H	H	H	H	H	H	H	H	H	H	H	H	H	H	H	H	H	H	C	C	C	C	C	H	H	H	H
Replicate B																																			
B1	H	H	H	H	H	H	H	H	H	H	H	H	H	H	H	H	H	H	H	H	H	H	H	H	H	H	C	C	C	C	C	H	H	H	H
B2	H	H	H	H	H	H	H	H	H	H	H	H	H	H	H	H	H	H	H	H	H	H	H	H	H	H	C	C	C	C	C	H	H	H	H
B3	H	H	H	H	H	H	H	H	H	H	H	H	H	H	H	H	H	H	H	H	H	H	H	H	H	H	C	C	C	C	C	H	H	H	H
B4	H	H	H	H	H	H	H	H	H	H	H	H	H	H	H	H	H	H	H	H	H	H	H	H	H	H	C	C	C	C	C	H	H	H	C
B5	H	H	H	H	H	H	H	H	H	H	H	H	H	H	H	H	H	H	H	H	H	H	H	H	H	H	C	C	C	C	C	H	H	H	H
B6	H	H	H	H	H	H	H	H	H	H	H	H	H	H	H	H	H	H	H	H	H	H	H	H	H	H	C	C	C	C	C	H	H	H	H
B7	H	H	H	H	H	H	C	H	H	H	H	H	H	H	H	H	H	H	H	H	H	H	H	H	H	H	C	C	C	C	C	H	H	H	H
B8	H	H	H	H	H	H	H	H	H	H	H	H	H	H	H	H	H	H	H	H	H	H	H	H	H	H	C	C	C	C	C	H	H	H	H
B9	H	H	H	H	H	H	C	H	H	H	H	H	H	C	H	H	H	H	H	H	H	H	H	H	H	H	C	C	C	C	C	H	H	H	C
B10	H	H	H	H	H	H	H	H	H	H	H	H	H	H	H	H	H	H	H	H	H	H	H	H	H	H	C	C	C	C	C	H	H	H	H
B11	H	H	H	H	H	H	H	H	H	H	H	H	H	H	H	H	H	H	H	H	H	H	H	H	H	H	C	C	C	C	C	H	H	H	H
B12	H	H	H	H	H	H	H	H	H	H	H	H	H	H	H	H	H	H	H	H	H	H	H	H	H	H	C	C	C	C	C	H	H	H	C
B13	H	H	H	H	H	H	H	H	H	H	H	H	H	H	H	H	H	H	H	H	H	H	H	H	H	H	C	C	C	C	C	H	H	H	H
B14	H	H	H	H	H	H	H	H	H	H	H	H	H	H	H	H	H	H	H	H	H	H	H	H	H	H	C	C	C	C	C	H	H	H	H
B15	H	H	H	H	H	H	H	H	H	H	H	H	H	H	H	H	H	H	H	H	H	H	H	H	H	H	C	C	C	C	C	H	H	H	H
B16	H	H	H	H	H	H	H	H	H	H	H	H	H	H	H	H	H	H	H	H	H	H	H	H	H	H	C	C	C	C	C	H	H	H	H
B17	H	H	H	H	H	H	H	H	H	H	H	H	H	H	H	H	H	H	H	H	H	H	H	H	H	H	C	C	C	C	C	H	H	H	H
B18	H	H	H	H	H	H	H	H	H	H	H	H	H	H	H	H	H	H	H	H	H	H	H	H	H	H	C	C	C	C	C	H	H	H	C
B19	H	H	H	H	H	H	H	H	H	H	H	H	H	H	H	H	H	H	H	H	H	H	H	H	H	H	C	C	C	C	C	H	H	H	H
B20	H	H	H	H	H	H	H	H	H	H	H	H	H	H	H	H	H	H	H	H	H	H	H	H	H	H	C	C	C	C	C	H	H	H	H
Replicate C																																			
C1	C	C	H	H	H	H	H	H	H	H	H	H	H	H	H	H	H	H	H	H	H	H	H	H	H	H	C	C	C	C	C	H	H	H	H
C2	C	C	H	H	H	H	H	H	H	H	H	H	H	H	H	H	H	H	H	H	H	H	H	H	H	H	C	C	C	C	C	H	H	H	H
C3	H	H	H	H	H	H	H	H	H	H	H	H	H	H	H	H	H	H	H	H	H	H	H	H	H	H	C	C	C	C	C	H	H	H	H
C4	H	H	H	H	H	H	H	C	H	H	H	H	H	H	H	H	H	H	H	H	H	H	H	H	H	H	C	C	C	C	C	H	H	H	C
C5	H	H	H	H	H	H	H	H	H	H	H	H	H	H	H	H	H	H	H	H	H	H	H	H	H	H	C	C	C	C	C	H	H	H	H
C6	H	H	H	H	H	H	H	H	H	H	H	H	H	H	H	H	H	H	H	H	H	H	H	H	H	H	C	C	C	C	C	H	H	H	H
C7	H	H	H	H	H	H	H	H	H	H	H	H	H	H	H	H	H	H	H	H	H	H	H	H	H	H	C	C	C	C	C	H	H	H	H
C8	H	H	H	H	H	H	H	H	H	H	H	H	H	H	H	H	H	H	H	H	H	H	H	H	H	H	C	C	C	C	C	H	H	H	H
C9	H	H	H	H	H	H	H	H	H	H	H	H	H	H	H	H	H	H	H	H	H	H	H	H	H	H	C	C	C	C	C	H	H	H	H
C10	H	H	H	H	H	H	H	H	H	H	H	H	H	H	H	H	H	H	H	H	H	H	H	H	H	H	C	C	C	C	C	H	H	H	H
C11	H	H	H	H	H	H	H	H	H	H	H	H	H	H	H	H	H	H	H	H	C	C	H	H	H	H	C	C	C	C	C	H	H	H	C
C12	H	H	H	H	H	H	H	H	H	H	H	H	H	H	H	H	H	H	H	H	H	H	H	H	H	H	C	C	C	C	C	H	H	H	H
C13	H	H	H	H	H	H	H	H	H	H	H	H	H	H	H	H	H	H	H	H	H	H	H	H	H	H	C	C	C	C	C	H	H	H	H
C14	C	C	H	H	H	H	H	H	H	H	H	H	H	H	H	H	H	H	H	H	H	H	H	H	H	H	H	H	H	H	H	H	H	H	H
C15	H	H	H	H	H	H	H	H	H	H	H	H	H	C	H	H	H	H	H	H	H	H	H	H	H	H	H	H	H	H	H	H	H	H	H
C16	H	H	H	H	H	H	H	H	H	H	H	H	H	H	H	H	H	H	H	H	H	H	H	H	H	H	C	C	C	C	C	H	H	H	H
C17	H	H	H	H	H	H	H	H	H	H	H	H	H	H	H	H	H	H	H	H	H	H	H	H	H	H	C	C	C	C	C	H	H	H	H
C18	H	H	H	H	H	H	H	H	H	H	H	H	H	H	H	H	H	H	H	H	H	H	H	H	H	H	C	C	C	C	C	H	H	H	H
C19	H	H	H	H	H	H	H	H	H	H	H	H	H	H	H	H	H	H	H	H	H	H	H	H	H	H	C	C	C	C	C	H	H	H	C
C20	H	H	H	H	H	H	H	H	H	H	H	H	H	H	H	H	H	H	H	H	H	H	H	H	H	H	C	C	C	C	C	H	H	H	H

Supplementary Figure S1c

Marker	1L	1R	2L	2R	3L	3R	4L	4R	5L	5R	6L	6R	7L	7R	8L	8R	9L	9R	10L	10R	11L	11R	12L	12R	13L	13R	14L	14LM	14LC	14RC	14R	15L	15R	16L	16R
Replicate A																																			
A1	H	H	H	H	H	H	H	H	H	H	H	H	H	H	H	H	H	H	H	H	H	H	H	H	H	H	C	C	C	C	C	H	H	H	H
A2	H	H	H	H	H	H	H	H	H	H	H	H	H	H	H	H	H	H	H	H	H	H	H	H	H	H	C	C	C	C	C	H	H	H	H
A3	H	H	H	H	H	H	H	H	H	H	H	H	H	H	H	H	H	H	H	H	H	H	H	H	H	H	C	C	C	C	C	H	H	H	H
A4	H	H	H	H	H	H	H	H	H	H	H	H	H	H	H	H	H	H	H	H	H	H	H	H	H	H	C	C	C	C	C	H	H	H	H
A5	H	H	H	H	H	H	H	H	H	H	H	H	H	H	H	H	H	H	H	H	H	H	H	H	H	H	C	C	C	C	C	H	H	H	H
A6	H	H	H	H	H	H	H	H	H	H	H	H	H	H	H	H	H	H	H	H	H	H	H	C	H	H	C	C	C	C	C	H	H	H	H
A7	H	H	C	H	H	H	H	C	H	H	H	H	H	H	H	H	H	H	H	H	H	H	H	H	H	H	C	C	C	C	C	H	H	H	H
A8	H	H	H	H	H	H	H	H	H	H	H	H	H	H	H	H	H	H	H	H	H	H	H	C	H	H	C	C	C	H	H	H	H	H	H
A9	H	H	H	H	H	H	H	H	H	H	H	H	H	H	H	H	H	H	H	H	H	H	H	H	H	H	C	C	C	H	H	H	H	H	H
A10	H	H	H	H	H	H	H	H	H	H	H	H	H	H	H	H	H	H	H	H	H	H	H	H	H	H	C	C	C	C	C	H	H	H	H
A11	H	H	H	H	H	H	H	H	H	H	H	H	H	H	H	H	H	H	H	H	H	H	H	H	H	H	C	C	C	C	C	H	H	H	H
A12	H	H	H	H	H	H	H	H	H	H	H	H	H	H	H	H	H	H	H	H	H	H	H	H	H	H	C	C	C	C	C	H	H	H	H
A13	H	H	H	H	H	H	H	H	H	H	H	H	H	H	H	H	H	H	H	H	H	H	H	H	H	H	C	C	C	C	C	H	H	H	H
A14	H	H	H	H	H	H	H	H	H	H	H	H	H	H	H	H	H	H	H	H	H	H	H	H	H	H	C	C	C	C	C	H	H	H	H
A15	H	H	H	H	H	H	H	H	H	H	H	H	H	H	H	H	H	H	H	H	H	H	H	H	H	H	C	C	C	C	C	H	H	H	H
A16	H	H	H	H	H	H	H	H	H	H	H	H	H	H	H	H	H	H	H	H	H	H	H	H	H	H	C	C	C	C	C	H	H	H	H
A17	H	H	H	H	H	H	H	H	H	H	H	H	H	H	H	H	H	H	H	H	H	H	H	H	H	H	C	C	C	C	C	H	H	H	H
A18	H	H	H	H	H	H	H	H	H	H	H	H	H	H	H	H	H	H	H	H	H	H	H	H	H	H	C	C	C	C	C	H	H	H	H
A19	H	H	H	H	H	H	H	H	H	H	H	H	H	H	H	H	H	H	H	H	H	H	H	H	H	H	C	C	C	C	C	H	H	H	H
A20	H	H	H	H	H	H	H	H	H	H	H	H	H	H	H	H	H	H	H	H	H	H	H	H	H	H	C	C	C	C	C	H	H	H	H
Replicate B																																			
B1	C	C	H	H	H	H	H	H	H	H	H	H	H	H	H	H	H	H	H	H	H	H	H	H	H	H	C	C	C	C	C	H	H	H	H
B2	C	C	H	H	H	H	H	H	H	H	H	H	H	H	H	H	H	H	H	H	H	H	H	H	H	H	C	C	C	C	C	H	H	H	H
B3	H	H	H	H	H	H	H	H	H	H	H	H	H	H	H	H	H	H	H	H	H	H	H	C	H	H	C	C	C	C	C	H	H	H	H
B4	H	H	H	H	H	H	H	H	H	H	H	H	H	H	H	H	H	H	H	H	H	H	H	C	H	H	C	C	C	C	C	H	H	H	H
B5	H	H	H	H	H	H	H	H	H	H	H	H	H	H	H	H	H	H	H	H	H	H	H	H	H	H	C	C	C	C	C	H	H	H	H
B6	H	H	H	H	H	H	H	H	H	H	H	H	H	H	H	H	H	H	H	H	H	H	H	H	H	H	C	C	C	C	C	H	H	H	H
B7	H	H	H	H	H	H	H	H	H	H	H	H	H	H	H	H	H	H	H	H	H	H	H	H	H	H	C	C	C	C	C	H	H	H	H
B8	H	H	H	H	H	H	H	H	H	H	H	H	H	H	H	H	H	H	H	H	H	H	H	C	H	H	C	C	C	C	C	H	H	H	H
B9	H	H	H	H	H	H	H	H	H	H	H	H	H	H	H	H	H	H	H	H	H	H	H	C	H	H	C	C	C	C	C	H	H	H	H
B10	H	H	H	H	H	H	H	H	H	H	H	H	H	H	H	H	H	H	H	H	H	H	H	H	H	H	C	C	C	C	C	H	H	H	H
B11	C	C	H	C	H	H	C	H	H	H	H	H	H	H	H	H	H	H	H	H	H	H	H	H	H	H	C	C	C	C	C	H	H	H	H
B12	H	H	H	H	H	H	H	H	H	H	H	H	H	H	H	H	H	H	H	C	C	C	H	H	H	H	C	C	C	C	C	H	H	H	H
B13	C	C	H	H	H	H	H	H	H	H	H	H	H	H	H	H	H	H	H	H	H	H	H	C	H	H	C	C	C	C	C	H	H	H	H
B14	H	H	H	H	H	H	H	H	H	H	H	H	H	H	H	H	H	H	H	H	H	H	H	H	H	H	C	C	C	C	C	H	H	H	H
B15	C	C	H	H	H	H	H	H	H	H	H	H	H	H	H	H	H	H	H	H	H	H	H	H	H	H	C	C	C	C	C	H	H	H	H
B16	C	C	H	H	H	H	H	H	H	H	H	H	H	H	H	H	H	H	H	H	H	H	H	H	H	H	C	C	C	C	C	H	H	H	H
B17	C	C	H	H	H	H	H	H	H	H	H	H	H	H	C	H	H	H	H	H	H	H	H	H	H	H	C	C	C	C	C	H	C	H	H
B18	H	H	H	H	H	H	H	H	H	H	H	H	H	H	H	H	H	H	H	C	H	H	H	H	H	H	C	C	C	C	C	H	H	H	H
B19	C	C	H	H	H	H	H	H	H	H	H	H	H	H	H	H	H	H	H	H	H	H	H	C	H	H	C	C	C	C	C	H	H	H	H
B20	H	H	H	H	H	H	H	H	H	H	H	H	H	H	H	H	H	H	H	H	H	H	H	C	H	H	C	C	C	C	C	H	H	H	H
Replicate C																																			
C1	H	H	H	H	H	H	H	H	H	H	H	H	H	H	H	H	H	H	H	H	H	H	H	H	H	H	C	C	C	C	C	H	H	H	H
C2	H	H	H	H	H	H	H	H	H	H	H	H	H	H	H	H	H	H	H	H	H	H	H	H	H	H	C	C	C	C	C	H	H	H	H
C3	H	H	H	H	H	H	H	H	H	H	H	H	H	H	H	H	H	H	H	H	H	H	H	H	H	H	C	C	C	C	C	H	H	H	H
C4	C	C	H	H	H	H	H	H	H	H	H	H	H	H	H	H	H	H	H	H	H	H	H	H	H	H	C	C	C	C	C	H	H	H	H
C5	H	H	H	H	H	H	H	H	H	H	H	H	H	H	H	H	H	H	H	H	H	H	H	H	H	H	C	C	C	C	C	H	H	H	H
C6	H	H	H	H	H	H	H	H	H	H	H	H	H	H	H	H	H	H	H	H	H	H	H	H	H	H	C	C	C	C	C	H	H	H	H
C7	H	H	H	H	H	H	H	H	H	H	H	H	H	H	H	H	H	H	H	H	H	H	H	H	H	H	C	C	C	C	C	H	H	H	H
C8	H	H	H	H	H	H	H	H	H	H	H	H	H	H	H	H	H	H	H	H	H	H	H	H	H	H	C	C	C	C	C	H	H	H	H
C9	H	H	H	H	H	H	H	H	H	H	H	H	H	H	H	H	H	H	H	H	H	H	H	H	H	H	C	C	C	C	C	H	H	H	H
C10	H	H	H	H	H	H	H	H	H	H	H	H	H	H	H	H	H	H	H	H	H	H	H	H	H	H	C	C	C	C	C	H	H	H	H
C11	C	C	H	H	H	H	H	H	H	H	H	H	H	H	H	H	H	H	H	H	H	H	H	H	H	H	C	C	C	C	C	H	H	H	H
C12	H	H	H	H	H	H	H	H	H	H	H	H	H	H	H	H	H	H	H	H	H	H	H	H	H	H	C	C	C	C	C	H	H	H	H
C13	H	H	H	H	H	H	H	H	H	H	H	H	H	H	H	H	H	H	H	H	H	H	H	H	H	H	C	C	C	C	C	H	H	H	H
C14	H	H	H	H	H	H	H	H	H	H	H	H	H	H	H	H	H	H	H	H	H	H	H	H	H	H	C	C	C	C	C	H	H	H	H
C15	H	H	H	H	H	H	H	H	H	H	H	H	H	H	H	H	H	H	H	H	H	H	H	H	H	H	C	C	C	C	C	H	H	H	H
C16	H	H	H	C	H	H	C	H	H	H	H	H	H	H	H	H	H	H	H	H	H	H	H	H	H	H	C	C	C	C	C	H	H	H	H
C17	H	H	H	H	H	H	H	H	H	H	H	H	H	H	H	H	H	H	H	H	H	H	H	H	H	H	C	C	C	C	C	H	H	H	H
C18	H	H	H	H	H	H	H	H	H	H	H	H	H	H	H	U	H	H	H	H	H	H	H	H	H	H	C	C	C	H	H	H	H	H	H
C19	C	C	H	H	H	H	H	H	H	H	H	H	H	H	H	H	H	H	H	H	H	H	H	H	H	H	C	C	C	C	C	H	H	H	H
C20	H	H	H	H	H	H	H	H	H	H	H	H	H	H	H	H	H	H	H	H	H	H	H	H	H	H	C	C	C	C	C	H	H	H	H

Supplementary Figure S2.

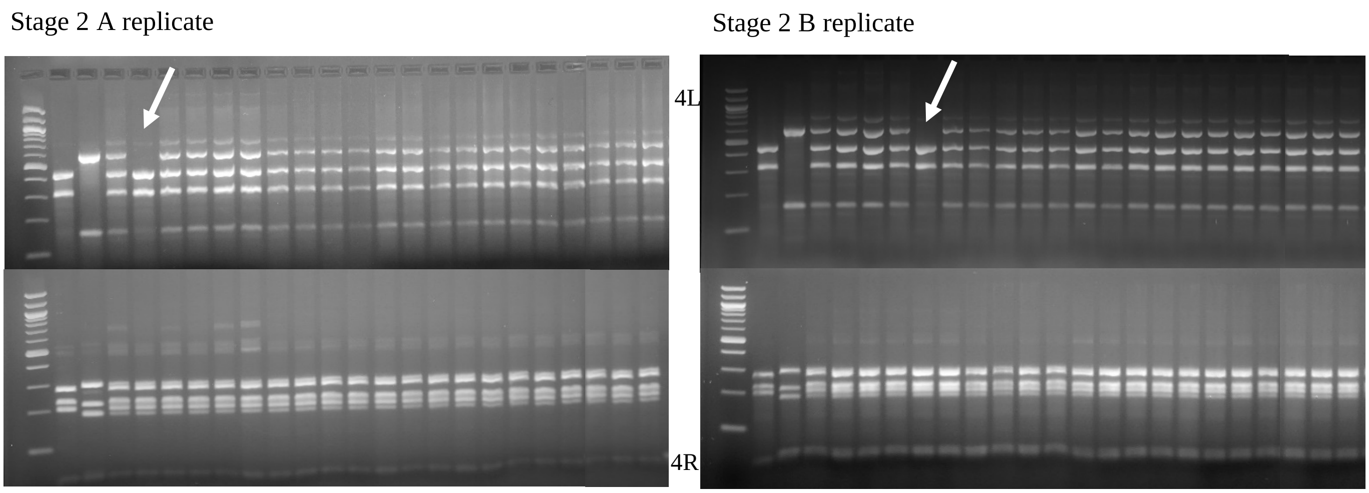

Supplementary Figure S3.

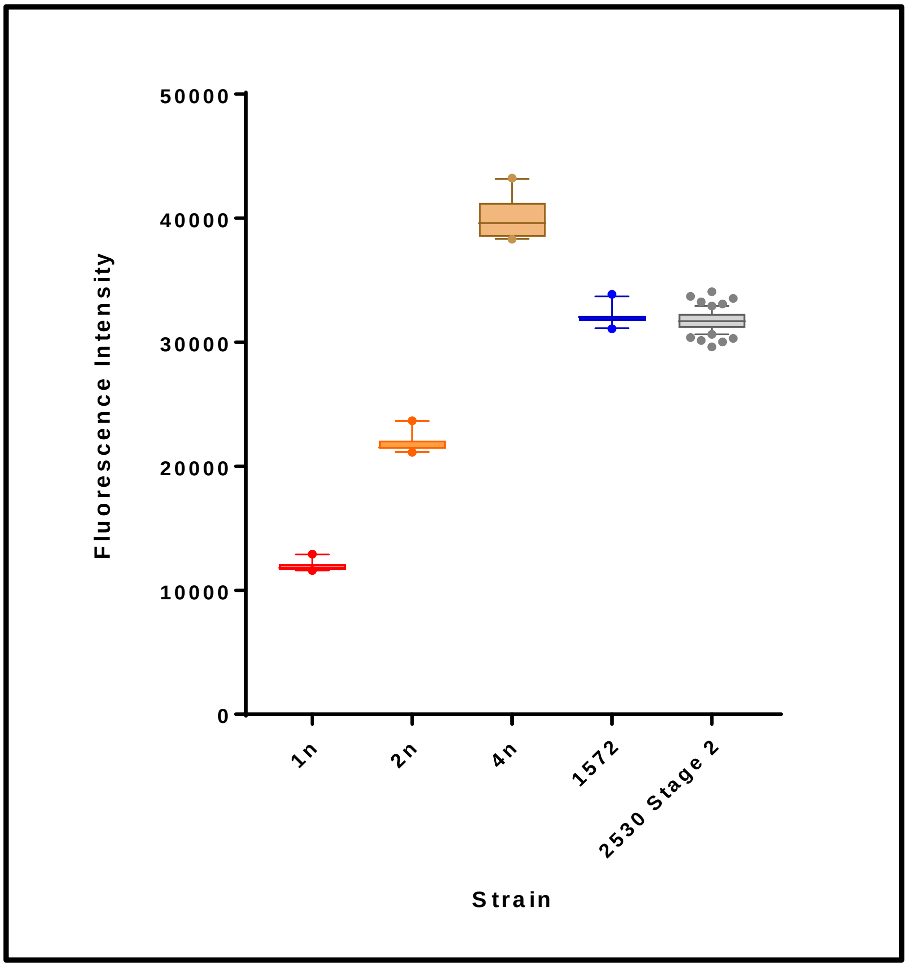

Supplementary Figure S4

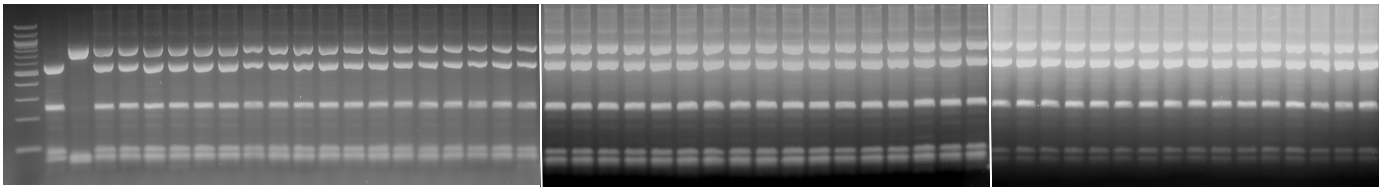

Stage 2A AWRI 1572 isolates #51-100

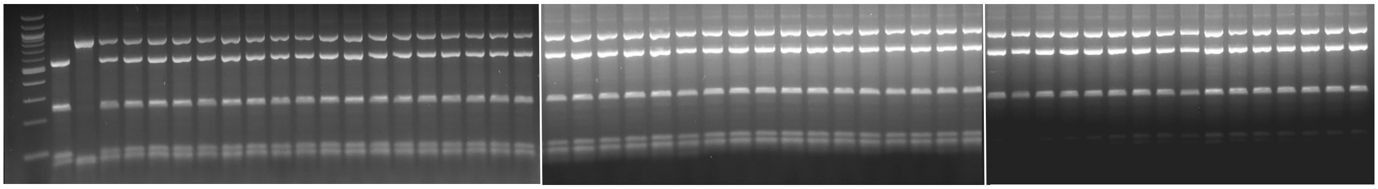

Stage 2B AWRI 1572 isolates #1-50

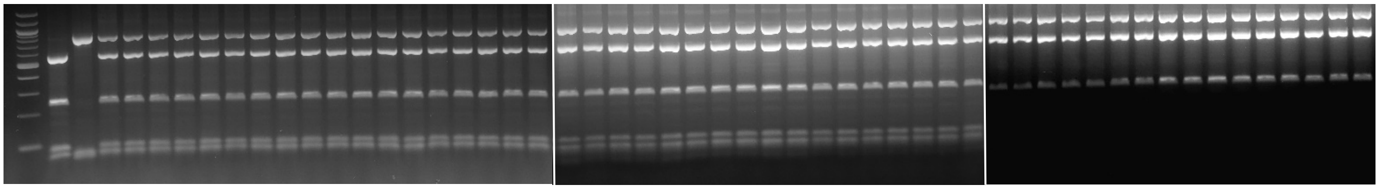

Stage 2B AWRI 1572 isolates #51-100

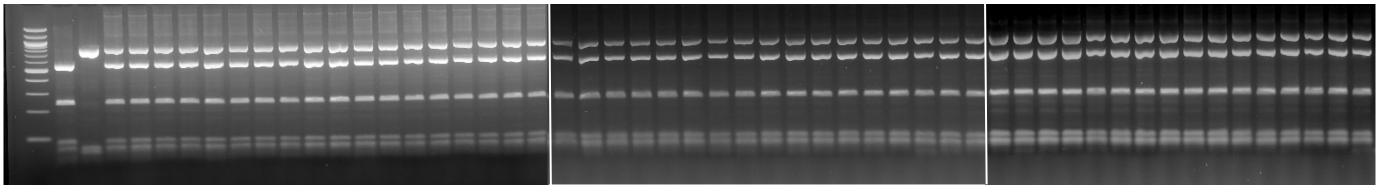

Stage 2C AWRI 1572 isolates #1-50

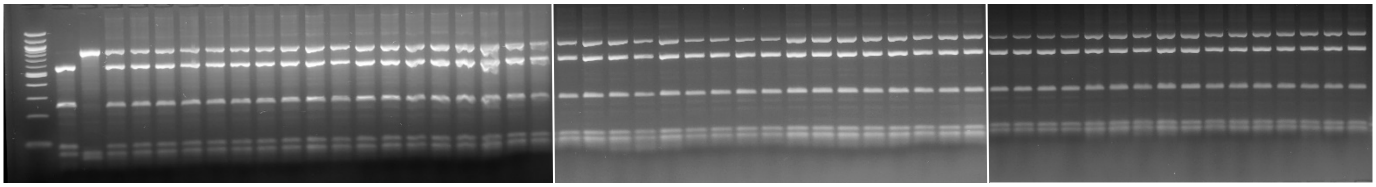

Stage 2C AWRI 1572 isolates #51-100

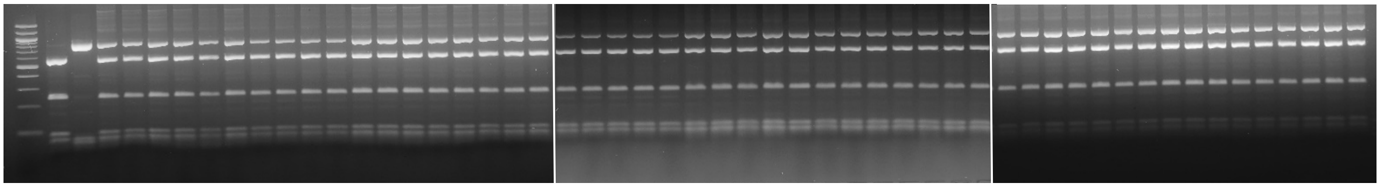

Supplementary Figure S5.

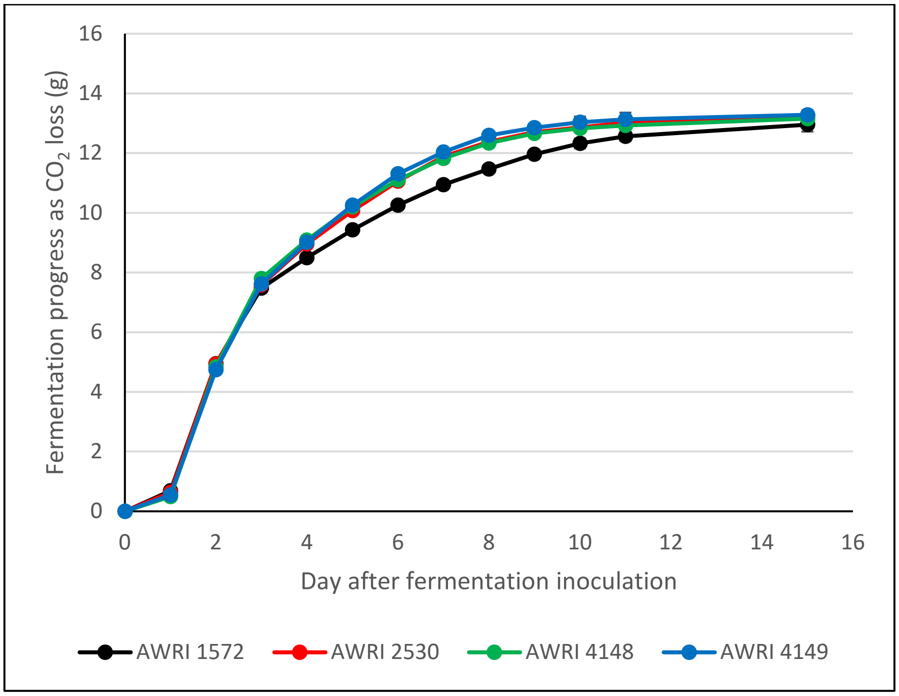

Supplementary Table S1.

Compound (g/L)	AWRI 1572	AWRI 2530
Chardonnay juice (225 g/L sugar)		
Glucose	0.00	0.00
Fructose	0.53 ±0.1 a	0.14 ±0.1 a
Glycerol	10.35 ±0.1 a	10.58 ±0.1 a
Ethanol*	14.9 ±0.1 a	15.1 ±0.1 a
Acetic acid	0.04 ±0.01 a	0.03 ±0.03 a
Succinic acid	4.25 ±0.02 a	4.45 ±0.04 a
Malic acid	4.33 ±0.04 a	4.46 ±0.03 a
Chardonnay juice (350 g/L sugar)		
Glucose	35.22 ±2.22 a	31.75 ±1.28 a
Fructose	88.59 ±2.38 a	86.57 ±3.43 a
Glycerol	14.12 ±0.82 a	14.65 ±0.35 a
Ethanol*	14.83 ±0.25 a	14.99 ±0.31 a
Acetic acid	0.30 ±0.02 a	0.27 ±0.05 a
Succinic acid	3.49 ±0.18 a	3.40 ±0.12 a
Malic acid	2.06 ±0.11 a	1.99 ±0.12 a
